# Review on Optical Methods Used to Characterize the Linear Birefringence of Polymer Materials for Various Applications

**DOI:** 10.3390/molecules28072955

**Published:** 2023-03-26

**Authors:** Dana Ortansa Dorohoi, Mihai Postolache, Cristina Delia Nechifor, Dan Gheorghe Dimitriu, Raluca Marinica Albu, Iuliana Stoica, Andreea Irina Barzic

**Affiliations:** 1 Faculty of Physics, ‘‘Alexandru Ioan Cuza’’ University of Iasi, 11 Carol I Blvd., RO-700506 Iasi, Romania; ddorohoi@uaic.ro (D.O.D.); dimitriu@uaic.ro (D.G.D.); 2 Faculty of Automatic Control and Computer Engineering, ‘‘Gheorghe Asachi’’ Technical University, 67 Dimitrie Mangeron Blvd., RO-700050 Iasi, Romania; mpostol@ac.tuiasi.ro; 3 Faculty of Machine Manufacturing and Industrial Management, ‘‘Gheorghe Asachi’’ Technical University, 67 Dimitrie Mangeron Blvd., RO-700050 Iasi, Romania; cd13_nechifor@yahoo.com; 4 Department of Physical Chemistry of Polymers, ‘‘Petru Poni” Institute of Macromolecular Chemistry, 41A Grigore Ghica Voda Alley, RO-700487 Iasi, Romania; stoica_iuliana@icmpp.ro

**Keywords:** optical polymers, linear birefringence, optical methods, applications

## Abstract

Optical polymers are recognized for their high transparency, raised flexibility, low cost, and good film-forming ability; hence, they introduce a multitude of benefits in a wide range of devices, such as information storage, displays, optical communications, and filters. Among the optical properties, birefringence is an essential parameter in practical cases that demand the control of the state of polarization of light. This review is focused on describing some fundamental and applicative aspects concerning the optical birefringence of the polymer materials. First, elementary notions depicting the phenomenon of light double refraction in macromolecular media are provided. Furthermore, the most relevant optical techniques to determine birefringence are reviewed by highlighting the working principle and mathematical basis for computing this parameter. Then, a series of investigations of optically birefringent polymers are described, summarizing the most utilized approaches to induce light double refraction in such materials. The selected results are analyzed in relation to the pursued applications. In the end, the future of this scientific domain is briefly presented by establishing the research paths that need further exploration. Moreover, the novel directions that could be formulated and might contribute to certain considerable advancements in the materials employed in the modern optical technologies are mentioned.

## 1. Introduction

Optical polymers (OPs) represent a category of engineering plastics of paramount importance in designing a variety of components for optical devices [1]. Substantial economy can be achieved by utilization of OPs for rendering aspheric and other complicated geometric surfaces, integrated parts with noncircular apertures, or elements of reduced sizes, which are expensive to manufacture in glass [2]. The introduction of OPs in all-plastic or hybrid systems imposes a deep understanding of their optical properties. Many optical characteristics, such as transparency, color, reflectivity, haze, and refractive index, are mainly influenced by two factors: the chemical structure and manner of material handling during the processing stage [2,3,4]. Related to the first aspect, the three-dimensional aspect typical for long macromolecular chains (composed of a multitude of subunits) determines the polarizability anisotropy. In the case of amorphous polymers, polarizability anisotropies of the structural units are compensated for since the macromolecular chains display a chaotic distribution [5]. Consequently, from a macroscopic point of view, the polymer can be regarded as being optically isotropic. However, this can be changed during the processing step if external factors produce orientation of the macromolecular chains. In such cases, the polymer exhibits a significant level of anisotropy in the optical properties. Special interest is attributed to the anisotropy of the refractive indices, which is quantified by an optical property called birefringence. Thick polymer layers having high birefringence can split the incident linearly polarized light into two linearly polarized rays (ordinary and extraordinary) by the phenomenon named double refraction. Many optical device producers are aimed at lowering birefringence by obstructing orientation of the macromolecules through various approaches, including an enhancement of the annealing time and temperature [6], blending with peculiar compounds [7], random copolymerization [8], or insertion of the anisotropic dopant [9]. In other applications, such as light modulators, optical storage discs, and phase retardation films, the polymer layer must display a considerable level of birefringence [10,11]. The latter might be induced by chain orientation via electrical forces [10], magnetic fields [12], polarized radiations exposure [13], acoustic waves [14], and mechanical forces [15,16]. The generation of optical birefringence in polymers by means of electric or magnetic fields is limited to materials that contain conductive or magnetic phases. Furthermore, the acoustically produced birefringence is observed only for liquid polymer systems. Therefore, the simplest ways to develop birefringence in polymers resides in the application of either radiation or mechanical forces. Hence, several studies have focused on the elucidation of the connection between birefringence and created orientation in polymer films by imposed deformation [15,17,18] or irradiation [19,20,21].

The birefringence of polymers is influenced by some factors, such as material composition [22], molecular weight [23], level/type of crystallinity [24,25], film thickness [23], and chain flexibility [26]. Moreover, the birefringence is dependent on the used experimental conditions, such as wavelength, temperature, and pressure [27,28,29,30].

In the depicted scientific context, this review focuses on the topic of optical birefringence since, in certain applications relying on polarized light, it is paramount to modify the polarization state, and this can be achieved by utilization of a quarter-wave plate or half-wave plate. In order to make such optical elements from polymers, it is essential to know the material’s birefringence and its variation with wavelength. Therefore, the aim of this work is to provide a comprehensive outlook on the birefringence phenomenon in polymer materials, which can give novel perspectives on the design of optical materials in relation to the desired optical anisotropy. It is important to acknowledge that polymer materials impose certain special requirements for the birefringence analysis in comparison to classical low-molecular-weight crystals: (a) the film preparation stage should be carefully finalized by avoiding bubble formation, inhomogeneity, and thickness variations; (b) the foils must have a flat surface because roughness determines incident light scattering; (c) many polymers are amorphous and, to gain molecular orientation, they require the action of external fields; thus, the birefringence can be tuned via the polymer sample processing conditions. The goal of the review is to present an overall image of the reports on the results related to birefringence of the polymer materials and to highlight the importance of the relation between the factors producing the macromolecular orientation (chain flexibility, drawing ratio, heating level, wavelength, and type of inserted additive) and the recorded birefringence. This optical parameter can be evidenced via several optical phenomena (refraction, interference, change of polarization state, etc.); thus, some relevant optical methods for birefringence characterization are detailed, namely, the interferometric method, compensatory method, channeled spectrum method, polarizing ellipse, refractometric method, and polarizing microscope method. All the presented techniques have a working principle described by specific mathematical approaches. Significant achievements on producing birefringence in polymer materials are depicted by presenting the external factor responsible for inducing the orientation of chains and the utilized conditions. The data are analyzed with regard to the polymer chemical structure characteristics, and the practical importance of each optically anisotropic system is shown.

## 2. Basic Aspects of Linear Birefringence

The linear birefringence, ∆*n*, of a uniaxial anisotropic layer is the difference between its main refractive indices (ordinary, *n_o_*, and extraordinary, *n_e_*), as visualized in Equation (1).

(1) $$\Delta n=n_{e}-n_{o}.$$

The ordinary refractive index is measured with linearly polarized radiations with the electric field vector perpendicular on the optical axis, while the extraordinary refractive index is measured with linearly polarized radiations with the electric field vector contained in the plane determined by the light propagation direction and the optical axis direction. The propagation direction for which the radiation speed does not depend on the polarization state is named the optical axis of the anisotropic medium. In the principal system of coordinates, the matrix of the refractive index has elements only on its diagonal. The axes of the principal system of coordinates, Oabc, named principal (main) axes, have the property that radiations propagating parallel to them do not change their polarization state. Uniaxial anisotropic media are characterized by $n_{a}=n_{b}=n_{o}$, and biaxial media are characterized by $n_{a}\neq n_{b}\neq n_{c}$.

In biaxial media, there are two directions for which the light propagation velocity does not depend on its polarization state, named optical axes. In the principal system of coordinates, Oabc, these media are characterized by three values of refractive indices, $n_{a}$, $n_{b}$ and $n_{c}$, for linearly polarized waves having their electric field vector perpendicular on the principal axes Oa, Ob, and Oc, respectively. By convention, the optical axes of the biaxial anisotropic media are considered as being contained in the principal plane Oac. The principal refractive indices of the biaxial medium can be determined using two samples cut from the same material, parallel to principal planes aOc and bOc, with linearly polarized waves having their electric vector perpendicular to the principal directions. Two measurements are made using linearly polarized waves with normal incidence on the surface of the sample. The techniques described for uniaxial anisotropic media can be used for biaxial media. In the two measurements, the principal refractive index corresponding to axis Oc is determined twice.

Generally, the birefringence of polymers can be imparted in several independent types, as shown in Equation (2) [31].

(2) $$\Delta n=\Delta n_{orientation}+\Delta n_{glass}+\Delta n_{form},$$

where $\Delta n_{orientation}$ denotes the birefringence caused by orientation of macromolecular chains, $\Delta n_{glass}$ is linked to the deformation with small strain, and $\Delta n_{form}$ arises from a nano- or microscale structure, including the micro-phase separation occurring in copolymers and nano-created features on the film surface. The glassy birefringence is negligible since it vanishes after cessation of stress. Molecular orientation and applied deformation have an insignificant effect on the form birefringence; however, the distinction in refractive indices among the components is relevant.

The main refractive indices are measured for linearly polarized radiations with the electric field intensity acting perpendicularly or parallel to optical axis (denoting the direction for which the light velocity does not depend on its polarization state). It is well known that linear birefringence is a dispersive parameter, being dependent on the light spectral composition. In the case of crystalline and amorphous polymers, the birefringence reflects the degree of orientation of crystalline regions or the alignment of chains under the influence of specific factors (i.e., solvent evaporation, thermal treatment, mechanical forces, acoustic waves, and electromagnetic fields).

When placing a birefringent layer between two polarizing filters (having their transmission directions at 90°), then the birefringence can be noted if the radiation is not passing parallel to the material’s optical axis. Polymers that exhibit two main refractive indices are considered uniaxial, whereas those having three main refractive indices are regarded as biaxial. Sometimes, the birefringence in a polymer is viewed as bands characterized by numerous colors. The principal reason for this can be explained as follows: when polarized radiation enters in the anisotropic medium, it is divided into rays (ordinary and the extraordinary rays), linearly polarized in two perpendicular directions. The ordinary ray is under the effect of the usual laws of refraction and moves with the same velocity unconstrained by direction, while the extraordinary ray is affected by the direction of movement. Upon exiting the anisotropic medium, the two rays keep the phase difference, and the polymer layers with variable thickness are viewed colored due to interference. The results of interference of ordinary and extraordinary rays depend on the wavelength of radiation, as well as on both linear birefringence and thickness of the polymer layer. In the case of polymer layers having constant thickness, the color separation due to their anisotropy can be evidenced in the channeled spectrum recorded using a spectrophotometer, when they are placed between two polarizing filters with crossed transmission directions [32].

The birefringence can be directly estimated or it can be computed by the difference shown in Equation (1), when the main refractive indices are separately measured. The main refractive indices can be measured by different experimental means.

The birefringence of molten polymer subjected to drawing deformation can be expressed by Equation (3).

(3) Δn=Cr×σ,

where *σ* is the drawing stress, and *Cr* denotes the stress-optical coefficient.

Similarly, the birefringence of a polymer found in a glassy state and subjected to mechanical stress is defined by Equation (4).

(4) Δn=Cp×σ,

where *Cp* represents the photoelastic coefficient.

The parameters *Cr* and *Cp* do not have identical values, while they can display distinct signs for particular polymers, which is an indicative that orientation behaviors at the molecular level are dissimilar in molten and glassy states [5]. The photoelastic coefficient is paramount for designing the majority of the polymer components that are incorporated in devices since they operate at temperatures corresponding to the glassy state of the material. The ∆*n* of polymers depicted by the *Cr* is not often kept as it is in optical devices even if they are processed by techniques that induce alignment of the chains (i.e., injection molding, extrusion, and drawing under heating) [5]. The long macromolecules are unwound and oriented in the molten phase under mechanical stress, after which they begin to relax in the next cooling stage, when conformational modifications appear. As a result, the alignment of macromolecular chains which continue to be in the glassy state produces birefringence. Thus, the birefringence observed in glassy polymers that retain the orientation level of the polymer chains is presented by Equation (5).

(5) $$\Delta n=f\times \Delta n^{0},$$

where $\Delta n^{0}$ denotes the intrinsic birefringence ascribed to the orientational birefringence for an extended chain (*f* = 1), while *f* refers to the orientation level of macromolecular chains.

This sort of birefringence is named orientational birefringence. Uniaxially drawn polymer layers are especially attained for orientational birefringence experiments. Intrinsic birefringence reflecting the inherent orientational birefringence characteristic of a material can be evaluated from the orientation degree, which is extracted from infrared spectroscopy or other techniques. The inherent birefringence of a polymer foil used in applications is described by intrinsic birefringence and the *Cp* parameter [33]. Form-related birefringence represents the birefringence determined by a periodic structure with dimensions under that of the wavelength, independent of optical anisotropy of the material [5].

## 3. Optical Methods for Linear Birefringence Evaluation

There are several experimental methods for determining the linear birefringence of polymer materials. In this section, certain significant optical techniques are summarized, namely, the interferometric method, compensatory method, channeled spectrum method, polarizing ellipse, refractometric method, and polarizing microscope method.

### 3.1. Interferometric Method

**Figure 1:**
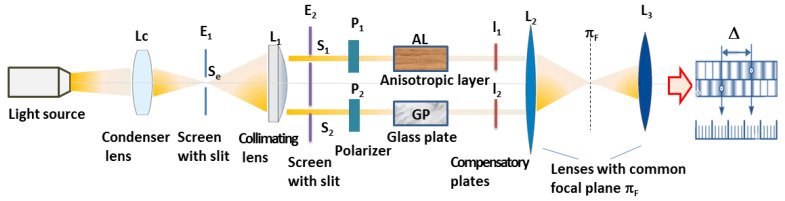
The scheme of Rayleigh interferometer used for linear birefringence evaluation.

Some of the methods devoted to estimate the linear birefringence of the anisotropic layers (ALs) are based on the interference. They involve the utilization of linearly polarized radiations and measurement of the pathway introduced in the two beams of the interferometer (Rayleigh interferometer) by the AL compared with the isotropic layer (usually a glass plate), which exhibits the value of the refractive index ranging between the ordinary and extraordinary refractive indices of the polymer AL and the same thickness as the AL [34]. In the two beams of the interferometer, two identical polarizing filters are introduced, as noted in the schematic representation for Rayleigh interferometer from Figure 1. The AL is placed in the measured beam, and a glass plate (GP) is put in the compensatory beam of the device. Two measurements are made with the transmission direction of the polarizing filter perpendicular to the measured beam or parallel to the optical axis of the AL. The values of the main refractive indices are determined from the shift between the central interference fringes (of the fixed and mobile fringe system) visualized in white light. Then, using the standardization graph of the interferometer for a given wavelength, the main refractive indices are estimated. For this purpose, Equation (6) is employed.

(6) $$n_{i}=n_{g}+\frac{k\lambda }{L},\;i=o,e,$$

where *k* is the order of the central fringe from the mobile fringes system corresponding to the central fringe from the fix system, *λ* is the wavelength used for standardization of the interferometer, and *L* is the thickness of the two layers (AL and GP) determining the fringe displacement. The indices *i* = *o*, *e*, and *g* refer to the AL with respect to the GP.

In Figure 1, the entry slit $S_{e}$ of the interferometer is directly illuminated by the condenser lens $L_{C}$, which gives the source image in the entry slit. The lens $L_{1}$ transforms the radiation in a parallel beam because its principal object focal plane is placed in the entry slit. Two beams are obtained by two identical slits $S_{1}$ and $S_{2}$. $P_{1}$ and $P_{2}$ are two identic polarizing filters, which transform the natural radiations in linearly polarized ones. AL is the anisotropic polymer foil introduced in the measured beam of the interferometer, and GP is a glass plate introduced in the compensation beam of the interferometer. The radiations are concentrated in the image principal focal plane of the lens $L_{2}$. The image can be analyzed with the lens $L_{3}$ having a magnifier role. Two systems of fringes are formed in the focal image plane of $L_{2}$. The fixed system of fringes is attained for radiations without a phase difference in the two beams. The mobile fringe system is due to radiations passing through the anisotropic and glass layers. The displacement between zero fringes (of the fixed and mobile fringe systems obtained in white light) is used to estimate the phase difference between the coherent radiations passing through the polymer layer and glass layer. In this method, the ordinary and extraordinary refractive indices are separately measured, and the birefringence is computed by their difference, as expressed in Equation (1).

### 3.2. Compensatory Method

**Figure 2:**
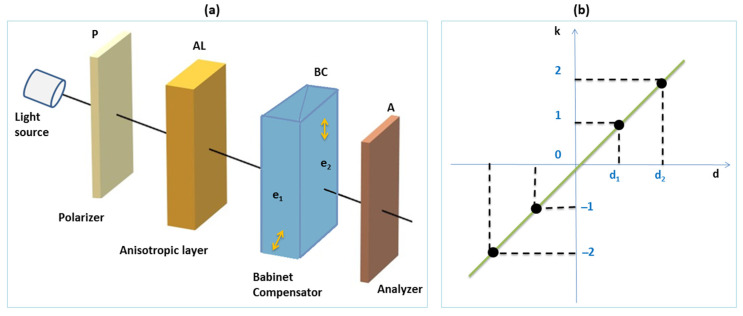
(**a**) Scheme of the device used with Babinet compensator; (**b**) standardization graph of the Babinet compensator.

The compensatory method asks for a compensatory anisotropic wedge, which can be moved as the light beam crosses it at different thicknesses (or for a compensator) to cancel the pathway between the ordinary and extraordinary light components passing through the anisotropic (polymer) layer (see Figure 2) [35].

The emergent light from the anisotropic layer passes through the compensatory wedge or through the Babinet compensator (BC). These devices introduce a phase difference equal to and with a contrary sign to that introduced by the AL.

The Babinet compensator is composed of two identical anisotropic prisms with small refractive angles. They are mounted with the refractive angle of the first based on the second with perpendicular optical axes (see Figure 2a). In this way, the ordinary ray from the first prism becomes extraordinary ray in the second. The prisms can be moved along their common surface, in order to be traversed by light at different thicknesses $e_{1}$ and $e_{2}$. The use of a Babinet compensator allows a very small variation of the compensatory layer ($e_{1}-e_{2}).$ The distance $e_{1}-e_{2}$ at the level of the light beam is measured with a micrometric tambour. A calibration (standardization) graph correlates the distance $e_{1}-e_{2}$ with the pathway $\Delta n^{\prime }\left(e_{1}-e_{2}\right)$ corresponding to the relative position of the two anisotropic prisms (Figure 2b).

In Figure 2, the device used for these kinds of measurements and the standardization graph of a Babinet compensator (from our laboratory) are given. The use of the BC allows a very small variation of the compensatory layer because its two prisms exhibit perpendicular optical axes. The prisms can be moved along their common surface, and their relative movement is monitored at the level of the light beam level. The phase difference $\Delta \psi^{\prime }$ introduced by the BC between the ordinary and extraordinary components of light is expressed by Equation (7), where $\Delta n^{\prime }$ is the birefringence of the prisms, and $e_{1}$ and $e_{2}$ are their thicknesses at the beam level.

(7) $$\Delta \psi \prime =\frac{2\pi }{\lambda }\Delta n^{\prime }\left(e_{1}-e_{2}\right),$$

The sense of the movement of the BC prisms must assure the opposite sign of the introduced phase difference, compared to that introduced by the AL (inserted between P and BC in Figure 2).

### 3.3. Channeled Spectrum Method

The channeled spectrum method is based on the interference of the ordinary and extraordinary components of the linearly polarized radiations crossing the AL. As a function of the linear birefringence and the thickness of the AL, the emergent radiations from the AL have different states of polarization. The phase difference Δψ between the components of light at the exit of AL depends on the birefringence Δn and on the thickness *L* of AL, as seen in Equation (8).

(8) $$\Delta \psi =\frac{2\pi }{\lambda }\Delta nL.$$

The phase difference at the exit of the AL determines the polarization state of the emergent light, as follows:

- The radiation keeps its linearly polarization state as at the entrance in AL, if Δψ=2mπ is an even number of π;
- Light is also linearly polarized, but has its azimuth changed in –*α*, if Δψ=(2m+1)π is an odd number of π;
- Light becomes elliptically polarized with the semiaxes of polarization ellipse parallel to the principal axes of AL, if the phase difference satisfies the condition Δψ=((4m+1)/2)π (the emergent light is circularly polarized for azimuth angles with an odd number of π/4);
- Light is elliptically polarized for the case 2mπ<Δψ<((4m+1)/2)π, but the polarization ellipse has its axes rotated relative to the principal axes of the AL.

**Figure 3:**
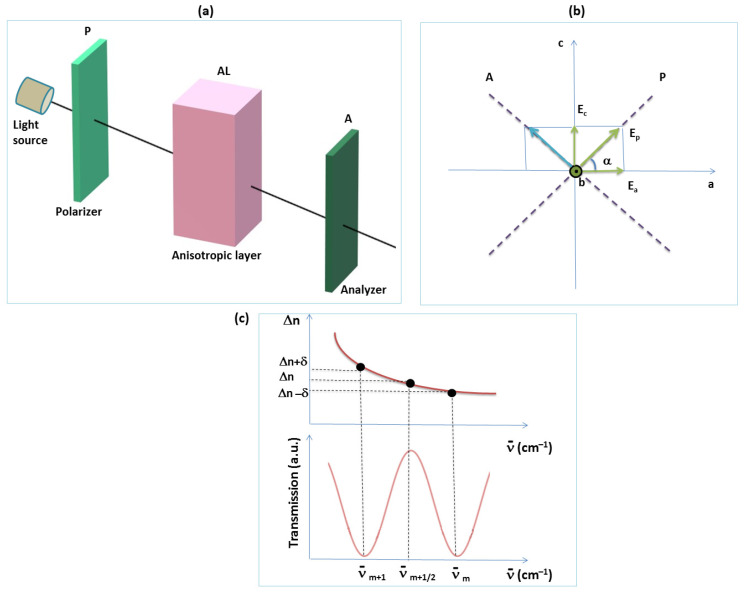
(**a**) Device used for obtaining channeled spectrum; (**b**) electric field vector at the entrance and the exit from the AL, for the first two conditions; (**c**) dispersive properties of birefringence of an anisotropic transparent material and representation of the two successive minima and a maximum from the channeled spectrum.

The device used to attain the channeled spectra is composed of two identical polarizing filers, and the polymer AL is placed between them, as indicated in Figure 3a.

**Figure 4:**
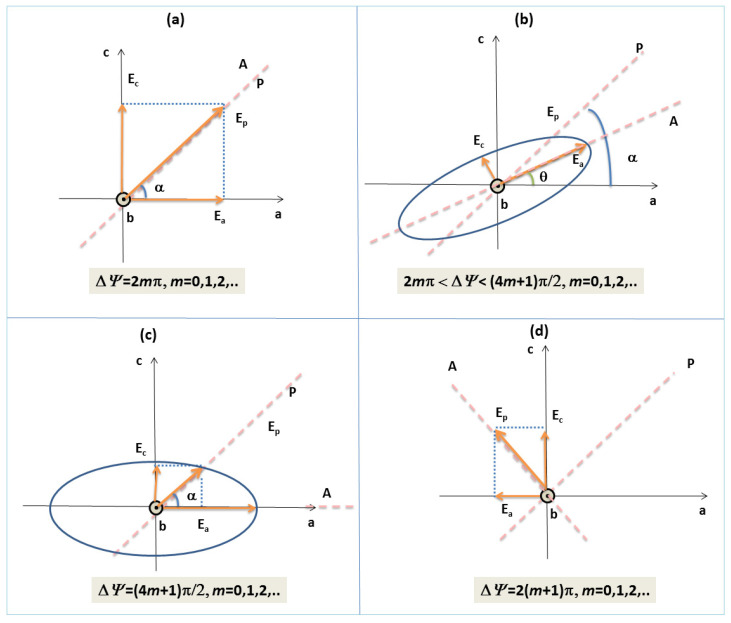
Polarization state at the exit of AL for different values of the phase difference between the ordinary and extraordinary radiations: (**a**) Δ*Ψ* = 2*m*π; (**b**) 2*m*π < Δ*Ψ* < (4*m* + 1)π/2; (**c**) Δ*Ψ* = (4*m* + 1)π/2; (**d**) Δ*Ψ* = (2*m* + 1)π.

Having in view the results mentioned before, when the AL is placed between two crossed polarizers, after the second polarizer (analyzer A), some radiations are characterized by null, maximum, or intermediate intensities as a function of the phase difference between their ordinary and extraordinary components (see Figure 4a–d). The spectral composition can be established if the device consisting of the two crossed polarizers and the AL is introduced in the measured beam of one spectrophotometer. A channeled spectrum consisting of successive nulls (channels) and maxima is obtained. The highest values for flux density of the maxima are obtained for azimuth angles of $\frac{\pi }{4}$.

The conditions of the channel appearance must take into consideration the dispersion of light by the AL sample (see Figure 3c). Let us consider Δe as being the birefringence of AL for the wavenumber $\nu_{m+\frac{1}{2}}$ corresponding to the maxima and δ as being the birefringence variation from this maximum and its neighbor minimum (Figure 3c). In such experiments, the dispersive parameter of birefringence is considered as being the same for the entire visible range. This supposition introduces a source of errors when the measurements are made in a large spectral range.

The conditions for the appearance of two channels and of the maximum between them are described by Equation (9).

(9) $$\{\begin{array}{c} 2\pi v_{m}\left(\Delta n-\delta \right)L=2m\pi \\ 2\pi v_{m+\frac{1}{2}}\Delta nL=\left(2m+1\right)\pi \\ 2\pi v_{m+1}\left(\Delta n+\delta \right)L=2\left(m+1\right)\pi \end{array}.$$

Equation (9) is written for a transparent AL whose birefringence satisfies the Cauchy law (decreases when the wavenumber decreases). Solving Equation (9), one obtains the solutions given in Equation (10).

(10) $$\{\begin{array}{c} m=\frac{v_{m}\left(v_{m+1}-v_{m+\frac{1}{2}}\right)}{v_{m+\frac{1}{2}}\left(v_{m}+v_{m+1}\right)-2v_{m}v_{m+1}}\\ \Delta n=\frac{1}{2L}\frac{v_{m+1}-v_{m}}{v_{m+\frac{1}{2}}\left(v_{m}+v_{m+1}\right)-2v_{m}v_{m+1}}\\ \delta =\frac{1}{2L}\frac{2v_{m+\frac{1}{2}}-v_{m}-v_{m+1}}{v_{m+\frac{1}{2}}\left(v_{m}+v_{m+1}\right)-2v_{m}v_{m+1}} \end{array}.$$

As it results from Equation (9), the birefringence Δn and its dispersion *δ* can be estimated only from the wavenumbers in the neighbor maxima and minima from the channeled spectrum of AL. In the case of polymer transparent layers of large thickness, when a great number of channeled spectra are obtained in the visible range, the birefringence and its dispersion can be evaluated with good precision for all components of the visible radiations.

### 3.4. Polarizing Ellipse Method

**Figure 5:**
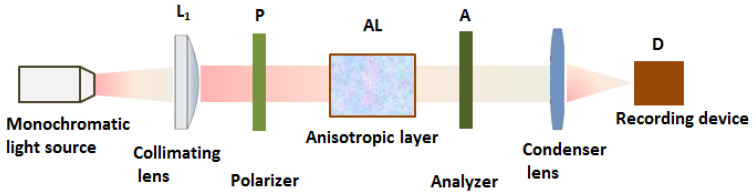
Schematic illustration of the device used for polarizing ellipse measurements.

This method—based on the determination of the rotation angle of the polarization ellipse relative to the principal axes of the AL—is a simple procedure consisting of the rotation of the analyzing polarizer around the light propagation direction until the maximum light intensity is obtained [36]. In this position, the transmission direction of the analyzer is parallel to the big axis of the polarizing ellipse. Then, a rotation of 90° determines the small axis of the polarization ellipse (by the smallest intensity of the light beam emerging from A). The device allowing birefringence determination by the polarizing ellipse is schematically represented in Figure 5.

Initially, the polarizers are crossed. The AL inserted between P and A changes the light polarization state of the linearly polarized light emergent from P. The relative positions of the transmission directions of the polarizers and the main axes of the polymer layer can be modified by rotating them around the light propagation direction. The system from Figure 5 works at a normal incidence of light and assures the light propagation along one principal direction of AL, differing from the optical axis (e.g., stretching direction in the case of polymer foils). The Ob axis is considered parallel to the light propagation direction (see Figure 4b). The azimuth angle (between the transmission direction of P and the Oa axis of the) is initially established. As a function of the value of Δψ introduced by AL, the emerging light from AL can have different polarization states (as in Figure 4a–d). Analyzer A can be rotated around the propagation direction until a null intensity is shown by detector D (if light is linearly polarized for conditions (a) and (b) in Figure 4) or the maximum of current intensity (when light is elliptically polarized in conditions (c) and (d) in Figure 4). In this way, it is possible to notice the corresponding values for angle *θ*. The angle of rotation *θ* (representing the angle between the principal axis of the AL and the big axis of the polarization ellipse) and the azimuth angle *α* are dependent, as depicted in Equation (11).

(11) tg2θ=cosΔψ tg2α,

where Δ, is the phase difference introduced by AL between the ordinary and extraordinary components of the light which crosses it. For known azimuth angle *α*, the phase difference introduced by AL determines the angle *θ*. Estimation of the birefringence of AL depends on the precision in angle determination. In order to increase the measurement precision, measurements at different azimuth angles α are performed, and the linear dependence in Equation (11) is graphically represented. The slope of the obtained line is the value of the function cosΔψ. The linear birefringence Δn of AL is then estimated using Equation (8). An angle gauge inclinometer is rigidly fixed on the mounts of the polarizer filters in order to increase the precision in estimating the angles α and θ. The flux density of the light emerging from analyzer A (see Figure 5) is measured by detector D in the external circuit of a diode with high sensitivity in the visible range. Monochromatic light is used in these types of measurements. The maxima and minima of the flux density are easily evidenced in the external circuit of the detector D. The polarizing ellipse method is very simple and can be used in laboratories where spectrophotometers, interferometers, or polarizing microscopes are not available for experiments. The method proposed and described in this review is somehow different from traditional ellipsometry, whereby the measurements are performed at a fixed incidence angle and the direction of the electric field vector (which does not occur in ellipsometry) is varied. In the polarizing ellipse method, the estimation of the birefringence depends on the precision in determination of the angles, namely, azimuth angle *α* and angle of rotation *θ*.

### 3.5. Refractometric Method

**Figure 6:**
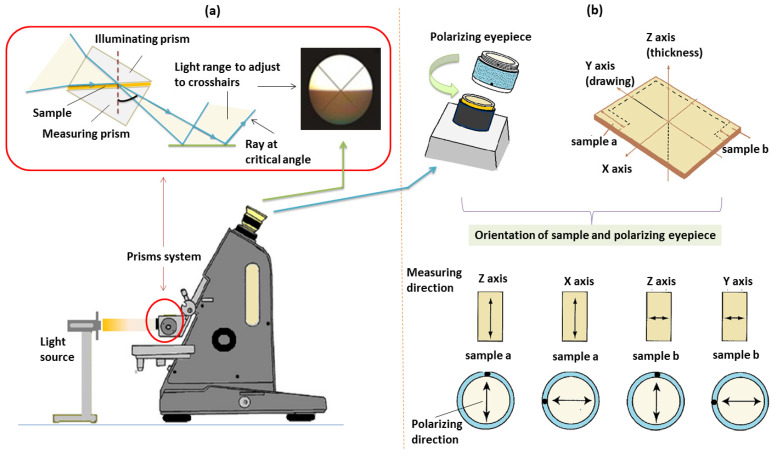
Representation of (**a**) the Abbe refractometer and (**b**) the principle of measuring birefringence of a material by polarized light refractometry.

The operating principle of an Abbe refractometer arises from the phenomenon of refraction, and it computes the refractive index of the examined material by utilizing Snell’s law. The principal elements of this device are the monochromatic light source, the mirror, the prism system, and the focusing telescope [37]. The incident radiation travels via the double prism containing the sample with the condition of having an angle of incidence at the interface under the critical angle of total reflection. Hence, the light reflected by the mirror passes via the illuminating prism having an upper rough (matted) surface (makes the radiation reaches the sample at a variety of angles inclusively parallel to the surface, which are essential for experiments). The radiation then reaches the AL and suffers refraction at the critical angle at the surface of the measuring prism; the critical rays produce the border between luminous and darker parts of the field examined with the telescope (see Figure 6a), which slides with the scale. The introduced compensator, namely, the Amici prism, is employed for avoiding the color fringes on the boundary line and for assessing the mean dispersion. With the help of the telescope, the position of the border between the bright and the dark areas is determined. The telescope reverses the image, such that the bright zone is up. Since the angle and the refractive index corresponding to the measuring prism are acquainted, the refractive index of the studied material is estimated by applying Snell’s law. According to Figure 6b, in order to perform birefringence measurements with an Abbe refractometer, it is mandatory to use a polarizing eyepiece. From the AL, two samples must be cut with distinct X- and Y-axes. Each sample is placed in the prism system, and then the polarizing eyepiece must be rotated to change the polarization direction, as illustrated in the Figure 6b. To evaluate the refractive index in the Z direction, the point mark must be placed at the topmost position. For the measurement along the X-direction, the point mark must be set sideways. When putting the sample b on the prism system, the polarizing eyepiece is moved to the topmost position, which gives the refractive index along the Z-axis. If the polarizing eyepiece is set sideways, the refractive index is determined along the Y-direction. Thus, the refractive index on the Z-axis can be estimated by two ways, and, if the data are different, it is recommended to adopt an average value.

### 3.6. Polarizing Microscope Method

**Figure 7:**
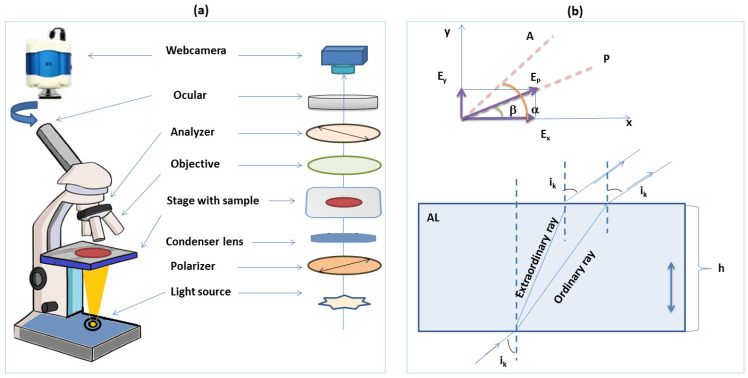
The representation of (**a**) polarizing microscope components and (**b**) electric field vector components along the polarizers, and scheme of the ordinary and extraordinary rays in the AL crossed orthogonal to the optical axis.

Another method for determination of birefringence for an anisotropic material is the use of a polarizing microscope, whose main components are depicted in Figure 7a [38]. When an AL, having *h* as thickness, is introduced between the polarizing filters (*A* and *P*), it creates a phase difference between the two components of the electric field vector. These components are able to recompose after the second polarizer plate, rendering a flux density that can be mathematically simplified as shown in Equation (12).

(12) $$\varphi_{A}=\varphi_{P}cos^{2}\Delta \psi \;/2.$$

Minima and maxima of the flux density are achieved when conditions from Equations (13) and (14) are fulfilled.

(13) Δψ=(2k+1)π, k=0, 1, 2…

(14) Δψ=2kπ, k=0, 1, 2…

Hence, in the case of Equation (12), null flux density is observed, whereas, for Equation (14), the flux density is $\varphi_{A}=\varphi_{P}$. Let us assume that the AL positioned among the polarizers (with transmission directions at 90°) is a uniaxial plate cut perpendicular to optical axis. The interference image produced in the focal plane of the device is affected by the phase difference created by the AL between the ordinary and extraordinary rays. In Figure 7b, the ordinary and extraordinary radiations coming from the polarized beam at incidence angle *i_k_* are illustrated. The pathway difference given by the AL among the ordinary and extraordinary counterparts denotes similarity in the case of directions having the identical incidence angle on the AL surface. The pathway difference Δ can be mathematically expressed as a function of the refractive index, which in turn depends on the incidence angle, as revealed by Equation (15).

(15) nef2=no2+(ne2−no2ne2)sin2ik.

According to Figure 7b, the pathway difference (Δ) can be expressed as in Equation (16).

(16) (Δ)h=no2−sin2ik(no2ne2)−no2−sin2ik=kλo2h, k=0, 1, 2…

Equation (16) allows the estimation of the main refractive indices and the birefringence by employing the experimental data for two rings (for two incidence angles ascribed to two consecutive maxima), when the thickness of the AL is known. Thus, *n_e_* and *n_o_* can be found by solving Equation (17).

(17) $$\{\begin{array}{c} \sqrt{n_{o}^{2}-\frac{n_{o}^{2}}{n_{e}^{2}}sin^{2}i_{k}}-\sqrt{n_{o}^{2}-sin^{2}i_{k}}=k\frac{\lambda_{0}}{2h}\;\left(k=1,\;2,\;3\ldots \right)\;\\ \sqrt{n_{o}^{2}-\frac{n_{o}^{2}}{n_{e}^{2}}sin^{2}i_{m}}-\sqrt{n_{o}^{2}-sin^{2}i_{m}}=m\frac{\lambda_{0}}{2h}\;\left(m=1,\;2,\;3\ldots ;m\neq k\right) \end{array}.$$

The radii of the rings are determined with accuracy so that the values of the refractive indices and the birefringence can be calculated.

## 4. Optically Birefringent Polymer Materials and Their Applications

The analysis of optically birefringent polymer materials is highly important when designing optical films for implementation in specific devices. The peculiarities of the chemical structure represent a key factor affecting the extent of the generated birefringence. Furthermore, the used technique to induce orientation of macromolecular chains can additionally influence the magnitude of this optical parameter because some polymers respond better to electromagnetic radiations, while others are more sensitive to the action of mechanical forces. Table 1 summarizes an overview of the results from the literature describing the birefringence of a set of selected polymers, along with the conditions used to acquire orientation.

It can be observed that the orientation level varies with the material’s structure and used experimental conditions. Moreover, the presented data reflect the implications of the malleability of the material on the created optical anisotropy. In particular cases, the incorporation of additive compounds has been proven to be useful for production of birefringent films as required in applications.

In this section, birefringence is discussed for the aforementioned polymer structures, and the data are presented in relation with the experimental method and the approach employed to produce the desired optical birefringence. The practical importance of the described polymer systems is reviewed.

### 4.1. Birefringence of Materials Based on Polyvinyl Alcohol

Polyvinyl alcohol (PVA) is a synthetic and water-soluble polymer, which is largely studied for its good film-forming characteristics, biodegradability, optical clarity, and mechanical stability [57]. The elevated transparency of PVA films makes them suitable for numerous applications, including in biomedicine for wound treatment [58]. The amorphous nature of this polymer makes it nonbirefringent from an optical point of view.

The double refraction phenomenon was evidenced in PVA samples stretched under temperature [39]. The characterization method used here involved a Babinet compensator for films with several thicknesses (1.3–2.46 mm). It was remarked that, as the film became thinner, the level of chain orientation, produced under mechanical force, became larger. Thus, at the biggest stretching degree of 3.9, the Δ*n* ranged from 0.0122 (highest thickness) to 0.0311 (smallest thickness). The irregular reduction in Δ*n* with the thickness might be due to the inability to produce a good alignment of the macromolecules from the films having a larger thickness. Birefringence of PVA was additionally studied using the polarizing ellipse method [36]. In this case, the sample processing was made in two stages: firstly, before completion of solvent removal a rubbing action was applied; secondly, after total solidification of the sample mechanical drawing was performed. When the stretching level was 1.90, Δ*n* had a value of about 0.0162481. Furthermore, drawing at 2.6 led to the Δ*n* enhancement up to 0.0261780. It seems that the rubbing procedure generates supplementary birefringence to the intrinsic Δ*n* observed for the pristine PVA layer. The data for the purely stretched sample, extracted using the polarizing ellipse approach, are in agreement with those achieved by the Babinet compensator technique.

Birefringence of PVA was evidenced by polarized microscopy recorded under variable temperatures [59]. Band-like folds rendering double refraction can be remarked in PVA hydrogel layers subjected to freezing–thawing treatment. Upon exposure to low temperatures, crystallites appear and favor crosslinking, resulting in the hydrogel state. When thawing, the condensed aqueous molecules are absorbed, determining hydrogel expansion and the occurrence of folds which display birefringence due to compressive stress. In another study involving the processing of polymers such as PVA under the form of fibers, the birefringence was monitored [60]. For the analysis of Δ*n*, the Pluta approach relying on an interference microscope enabling to control wavefront shear [61] was used. It was reported that, at 546 nm and 550 nm, the values of birefringence of PVA fibers were 0.039 and 0.038, respectively.

On the other hand, it was demonstrated that exposure to UV and gamma radiations in combination with stretching also affect the birefringence [40]. By employing the compensatory method, it was shown that the unexposed films displayed birefringence enhanced by mechanical stretching. Upon irradiation, the PVA samples exhibited diminished birefringence, which was slightly augmented for larger UV exposure periods, but did not exceed the Δ*n* values of pristine samples. Moreover, for a constant stretching ration, the ordering level from the gamma ray-exposed PVA film increased beyond the Δ*n* of the nonirradiated counterpart. The slope of the plots of Δ*n* versus stretching degree showed modifications in the alignment mechanisms of macromolecules from the stretched foils. Thus, a lowering of the slope revealed the saturation of Δ*n* at stretching degrees over that value. The slope was diminished owing to the process of relaxation and hindering of chain ordering. The level of stretching degree was reduced with irradiation time, meaning that photo-oxidation and photo degradation phenomena occurred, and the macromolecules tended to relax more rapidly. Such results are meaningful for radiation shielding and for space applications. A similar investigation involved exposure to microwaves of the PVA aqueous solution followed by film drawing [41]. Several absorption energies were applied to the samples (0.64 kJ/mL, 0.99 kJ/mL, and 1.61 kJ/mL). At the stretching degree of 3, the birefringence of irradiated films ranged from 0.006 to 0.0183 as the absorption energy was enhanced. From the obtained slopes for Δ*n* against the stretching level, it was assumed that saturation of the chain alignment for these foils was not accomplished for drawing ratios under 4. The birefringence investigations indicated that the optical anisotropy of microwave-treated samples was smaller than that of the pristine samples.

The double refraction phenomenon can be observed in PVA when it is exposed to acoustic waves [14]. A stationary Δ*n* of PVA solutions in water was noted at room temperature, increasing linearly with the solid concentration. The adopted orientation of the macromolecules was activated by the presence of the acoustic waves and could be imposed via the torque, which led to the polymer aligning parallel to the external acoustic field. In solution, the PVA chains lost their isotropy and became isolated to a certain extent by the water molecules. The acoustic waves produced deformation of the original form of the macromolecules, determining additional anisotropy, and this phenomenon was amplified by the addition of PVA to the system. The values of Δ*n* of PVA solutions increased at higher ultrasonic frequencies, due to the fact that the water and PVA molecules displayed distinct translational velocities. Upon frequency increasing, the translation of the overlapped macromolecules was more impeded with respect to water. The translational velocity dissimilarity impacted by frequency produced larger radiation pressure; therefore, birefringence was augmented at high frequency. Moreover, Δ*n* values increased with ultrasonic intensity.

Samples with better orientation are ideal for use in biomedical fields to release drugs. Accordingly, some investigations were devoted to examining (using several characterization methods) the birefringence induced in PVA foils containing certain additives. The solid materials were subjected to orientation by different means. For instance, donepezil was introduced in PVA, and the prepared system was mechanically drawn [45]. The optical analysis method involved a Babinet compensator. It was noted that insertion of this drug favored the alignment of PVA chains via physical interactions with the functional groups of the system components. A linear increase in generated birefringence was attained. At a stretching degree of 1.05, the birefringence was 0.005, whereas at a 1.4 stretching degree, Δ*n* was 0.025. It can be stated that the mechanical force produced drug arrangement in the PVA matrix, as indicated by the optical analyses.

The double refraction in PVA can be influenced by the presence of other compounds, such as poly(N-vinyl pyrrolidone) (PVP) [62]. The uniaxial deformation of this polymer mixture led to optical anisotropy, which was analyzed via polarizing microscopy combined with a Berek compensator. The evolution of the sample orientation upon mechanical drawing was slightly impeded by augmenting the amount of PVP. When the molecular weight of PVA was lower than that of PVP, a larger molecular orientation was attained in comparison to the case of the larger molecular weight of PVA. The magnitude of Δ*n* of the stretched foils was enhanced when the PVA amount prevailed in the system, potentially modifying its sign at a particular fraction. The delimiting PVP fraction was noted to be smaller in the system containing PVP of larger molecular weight than PVP. This aspect can be attributed to an effect of Δ*n* compensation as a result of variation in the sign of Δ*n* of the stretched PVP and PVA. For systems with PVP of lower molecular weight, the largest Δ*n* of 24 × 10^−3^ was noted at 300% elongation at a mixing ratio 90 PVA/10 PVP, while the smallest Δ*n* of −0.24 × 10^−3^ was noted for 10 PVA/90 PVP at 900% elongation. The other system with high-molecular-weight PVP displayed the largest Δ*n* of 22.6 × 10^−3^ at 260% elongation at a mixing ratio of 90 PVA/10 PVP, whereas the smallest Δ*n* of −3.6 × 10^−3^ corresponded to 20 PVA/80 PVP at 820% elongation.

The molecular orientation of spun PVA fibers can be improved by the incorporation of iodine [63]. It was noted that a small quantity of iodine induced greater stretching ability and alignment because the interactions among the polymer chains were diminished. The double refraction was examined on an Interphako interference microscope. The experiments revealed that iodine doping enhanced Δ*n* from 52 × 10^−3^ to 49 × 10^−3^ for the neat sample, in similar drawing conditions. This was caused by the better ability of chain slippage in the amorphous zones favored by iodine. X-ray analysis indicated that crystal orientation was similar regardless of the dopant presence in PVA. Coupling this information with birefringence data, it seems that the molecular alignment during drawing of doped PVA is manly influenced by the high orientation of the amorphous component of the sample. Such fibrous materials are interesting for industrial and technical applications.

The molecular ordering in PVA fibers subjected to several draw ratios was reported in [64]. The data were collected on a Zeiss polarization microscope connected to an Ehringhaus compensator. It was found that the intrinsic Δ*n* corresponding to the amorphous phase was 79 × 10^−3^, larger than that characterizing the crystalline part of PVA fiber (52 × 10^−3^). As expected, the values of Δ*n* were augmented as the drawing level increased. At a draw ratio of 1, the Δ*n* magnitude was quite large, perhaps owing to effects of pre-stretching (undeformed fibers had Δ*n* = 26 × 10^−3^), including the impact of stretching under temperature, which rendered the molecular orientation of the sample.

In a different study [65], the drawability of PVA solution in a dimethyl sulfoxide/water mixture was tested in conditions of gelation and crystallization. It was demonstrated that the sample capacity of drawing was limited by the ratio of the solvent mixture, as well as by the heating conditions. The largest variations in birefringence were observed as a function of solvent compositions, while the crystallinity level, degree of polymerization, and the concentration of PVA in solution produced less variation of this optical parameter. The biggest Δ*n* of 48 × 10^−3^ was found at 70/30 Me_2_SO/H_2_O, while the smallest Δ*n* of 40.4 × 10^−3^ was recorded at 100/0 Me_2_SO/H_2_O.

Another relevant case study was devoted to PVA/pyridinium ylide films, which were mechanically stretched; the birefringence was evaluated by utilizing a polarizing microscope coupled to a compensatory wedge [42]. It was observed that, depending on the type of introduced ylide structure, the birefringence was different, regardless of the imposed stretching level. For example, for an ylide having –CO_2_C_2_H_5_ as the terminal group, the birefringence varied from 0.0011 (0.12 stretching degree) to 0.032 (0.35 stretching degree). When the ylide displayed –CO_2_C_2_H_5_ and –CONHC_6_H_5_ as the terminal groups, the birefringence was larger, i.e., 0.0125 (0.12 stretching degree) and 0.033 (0.41 stretching degree). The observed linear variation between the foil birefringence and the degree of order in the PVA-based films is useful for optical filter devices. The birefringence study of PVA containing phthalazinium ylide was performed [43]. For stretching levels under 3.5, the birefringence increased linearly with the imposed deformation, whereas, above this value, a saturation tendency in the macromolecular orientation took place, and Δ*n* did not exhibit such a wide range. The Δ*n* of the ylide-doped PVA samples was larger than that of the neat polymer up to a stretching level of 3.5. Beyond this poiint, Δ*n* values were similar to those registered for the pristine PVA. At a stretching degree of 3, the birefringence ranges were as follows: 0.019 (phthalazinium-di-benzoyl-methylide) < 0.022 (6-methyl-phthalazinium- di-benzoyl-methylide) < 0.024 (6-methyl-phthalazinium-cyano-carbethoxy-methylide). The most pronounced birefringence of 0.0254 (stretching degree of 5.5) was noted for the PVA films containing 6-methyl-phthalazinium-cyano-carbethoxy-methylide. These data are important for polarizing filters. In another study, a comparison of pure PVA and PVA doped with cycloimmonium ylide was conducted [44]. The calculated birefringence corresponding to the dyed PVA samples was augmented with regard to the neat polymer. This means that the ylide dipolar molecules provided a relevant contribution to the system alignment under drawing. The Δ*n* of the studied foils depended of the order degree, which could be described by a second-order polynomial expression. Depending on the chemical structure of the used dye, the dipolar additive of cycloimmonium ylide favored the orientation of the side-groups of the polymer. The largest birefringence of 0.0275 (at a stretching ratio of 5) was recorded for PVA doped with the ylide having the 3-(p-phenyl)-pyridazinium heterocycle. Materials based on PVA and Congo red dye were analyzed from a double refraction point of view in [46]. The stretched films were characterized by means of the Babinet compensator technique. The dye component was incorporated in various concentrations of PVA (2%, 3%, 6%, and 7%. At a fixed stretching level, it could be noted that the addition of dichroic molecules determined an increase in the sample alignment under a mechanical field. This was confirmed by the computed values of the ordering degree at variable stretching. At the largest stretching of 3, birefringence reached the value of 0.08. These materials present adequate properties for dye-related applications.

The birefringence of PVA was also studied in the presence of several dye molecules (methyl orange indicator, Congo red, phenol red, and methyl red) for waveguide purposes [66]. The results attained using the prism coupling approach showed that the dyed foils and the pristine ones displayed zero birefringence. The presence of dyes influenced certain basic waveguiding factors, but the optical experiments revealed that birefringence was not changed in the absence of external stress.

A distinct approach for inducing double refraction PVA resides in surface adaptation under mechanical fields [67]. A combination of rubbing (prior to complete solidification) and stretching was employed, and the birefringence was estimated with the help of a Rayleigh interferometer. The rubbing step followed by drawing created additional alignment of the macromolecules, and this could be seen from the bigger Δ*n* in comparison to the non-rubbed and stretched sample. The results were compared to those acquired using the Babinet compensator, and slightly higher values were given by the interferometric method at the same deformation level. Moreover, at the largest drawing ratio of 4, the birefringence of non-rubbed samples was 0.0172, while that of the rubbed films was 0.0365 (using the interferometer); in contrast, Δ*n* ranged from 0.0161 to 0.0355 using the compensator device. A higher birefringence was found to be compatible with higher adhesion of the nematic compound placed on the polymer support. This determined better orientation of the liquid crystal on the birefringent polymer, as demanded in display applications.

Introduction of azo-derivatives in PVA films under stretching also created optical birefringence [68], as examined with the Babinet compensator instrument. In this case, the films suffered a similar mechanical treatment (stretched or rubbed and stretched). The attained values of Δ*n* for these materials varied between 1 × 10^−2^ and 3 × 10^−2^. The type of inserted azo-dye in the system created distinct levels of ordering under mechanical treatment. It was shown that *p*-nitrophenol led to greater birefringence than nitrobenzene or *p*-nitroaniline. Thus, it appears that the chemical structure of the azo-compound and the applied mechanical deformation procedure allow controlling the double refraction level in polymer materials, which is useful for interference filter manufacturing.

On the basis of the exposed studies on PVA, it can be stated that this nonbirefringent polymer becomes anisotropic under the action of (a) a mechanical field applied during processing under the form of fibers or stretched films, (b) variable temperature (freezing/thawing treatment), (c) UV and gamma radiation, (d) acoustic waves, and (e) dissolution in solvent mixtures. Depending on the incident radiation energy, the irradiated PVA birefringence can be higher or lower than that observed for the pristine polymer. When using a solvent mixture for obtaining PVA films, the composition has proven to be a decisive factor for the final film’s birefringence. Moreover, the presence of additives, such as drugs, dyes, or other polymers, has been shown to vary the birefringence in comparison to neat PVA material. The structural features of the additive have been found to affect the achieved level of molecular ordering of the PVA system subjected to external factors, especially under mechanical deformation. It seems that insertion of dichroic molecules (dye substances) or iodine favors PVA chain slippage during mechanical drawing, and the recorded birefringence is larger. Birefringent PVA layers are useful in many areas, ranging from drug release and optical filters to alignment layers for displays.

### 4.2. Birefringence of Materials Based on Polyethylene Terephthalate

Polyethylene terephthalate (PET) is a transparent thermoplastic compound which belongs to the polyester family. This polymer can be made into a variety of shapes, and it is recommended for many applications. In a recent report [47], it was demonstrated that PET films are characterized by optical birefringence. The channeled spectrum method was employed to compute the variation with wavelength of this parameter. The number of channels in the spectrum was influenced by both the level of anisotropy and the thickness of the examined material. A reduction in birefringence was noted with increasing wavelength of the incoming ray, from 0.0644 at 400 nm to 0.0487 at 750 nm. Furthermore, the dispersive parameter was also estimated, and it was revealed that it was not a characteristic of the PET sample. Its variation with light energy indicated that the made assumption of a constant value is not entirely correct in the visible domain. In any case, given its small magnitude (~100 times lower than Δ*n*), it could be considered that the reported data displayed high precision.

An interesting study [49] attempted to clarify the relation between stress and optical performance of PET. The material gained molecular ordering through simultaneous biaxial stretching. To determine the mechano-optical characteristics, a lab-made device was used. It consisted of a high-speed video camera connected to an instrumented biaxial deformation system to monitor the pattern printed on the polymer layer. Utilizing such an experimental system, it was possible to register the birefringence values in real time. The measurements led to the conclusion that there are several regimes depicting the relationship between deformation and birefringence. In the first zone, the birefringence ranged linearly with stress. Here, the birefringence was around 0.02 for a true stress of 4 MPa and biaxial stretching at 95 °C. Then, the second domain was noted, where the relationship was almost linear with a sharper slope, whereas, in the third zone, the relationship became nonlinear. When significant stretching rates were employed, another domain was remarked, where the stress was enhanced but the birefringence remained nearly constant (Δ*n* = 0.12 at true stress of 14 MPa). This situation took place when the macromolecular chains reached finite extensibilities. At small deformation rates, this zone was not noticed because large relaxation motions prevailed over the orientation effects. The departure from the linear stress optical rule concurred with the appearance of stress-generated crystallization, as indicated by the offline recordings. Such results are essential for nonlinear optic devices.

Another study dealt with the birefringence of PET fibers generated by hot multistage drawing [48]. The experiments were carried out on a Zeiss polarizing microscope coupled with a tilting compensator. PET samples with either amorphous or crystalline characteristics (different degrees of order) were selected. The birefringence of PET with randomly distributed chains is 0.0029. At a crystallinity of 41% and a drawing ratio of 4.68, Δ*n* became 0.17, whereas, at 49% and a drawing ratio of 6.24, Δ*n* reached 0.2069. Under hot multistage drawing, imposed deformation and applied temperature led to considerable modifications of the optical and structural properties of PET fibers. The birefringence displayed a linear enhancement up to a high draw ratio with increasing draw ratio. This was ascribed to considerable chain extension during the mechanical force action with reduced chain relaxation under applied temperature. These data are important for making phase retardation films.

A recent paper [50] presented the strain-optical behavior for the same polymer, where the birefringence was estimated by means of Mueller matrix ellipsometry. The PET samples were biaxially stretched during film preparation. The computed values of the strain-induced birefringence were affected by the orderly orientation of an amorphous and crystalline zone along the mechanical force, which was typically enhanced for larger strains when tensile loading acted in the transverse direction. In contrast, the magnitude of birefringence decreased with the strain augmenting along the machine direction. A linear variation between birefringence and orientation was attained; for a strain of 0.05, Δ*n* was 0.045, whereas, for a strain of 0.2, Δ*n* reached 0.058.

Analyzing the case studies on PET, it appears that this material is an anisotropic material. The birefringence of this optical medium decreases with increasing light wavelength. Under biaxial stretching, PET displays several zones in which birefringence ranges differently with the applied stress. The influence of the amorphous or crystalline characteristics of PET is emphasized under variable temperature drawing, evidencing that the degree of crystallinity enables a certain level of deformation under stretching that generates a linear enhancement of birefringence. Such aspects are critical for designing components relying on the optical retardation phenomenon.

### 4.3. Birefringence of Materials Based on Polyimides

Polyimides (PIs) are largely reported as being thermostable polymers with good mechanical and optical properties. The optical clarity of these materials is related to the level of chain conjugation; hence, for elevated transparency, monomers with low polarizability are preferred. Moreover, it was demonstrated that, depending on the degree of imidization and the used cyclization procedure, PIs could gain optical birefringence [51]. When the solvent was quickly removed during film solidification under a certain temperature, Δ*n* increased regardless of the used imidization route. After the imidization stage, the product displayed considerable intrinsic birefringence in comparison to its precursor. Chemical imidization led to PI materials with larger in-plane orientation compared to compounds attained by thermal imidization. The introduction of aliphatic sequences in the PI structure determined a diminishment of the molecular charge transfer interactions [52]. This induced a reduction in PI chain stacking such that the disorder level in the material was increased, as reflected in the lower birefringence.

Optical anisotropy can be generated in PIs by various means. One of the first reported methods was based on the anisotropy observed in the thermal expansion coefficients of the supports with the role of drawing force [69]. A fluorinated PI was placed on several supports (calcite, silicon, quartz, and lithium tantalate), and then peeled from them at the drying stage. The optical behavior of these PI films was examined. The data showed that the silicon support induced a very low birefringence (under 0.001) because this material is characterized by an isotropy of physical properties. On the other hand, the substrates exhibiting a broader anisotropy of thermal properties were capable of generating a higher extent of anisotropy. This was confirmed by the measured birefringence values with a rotating Nicol prism at 633 nm. The macromolecular PI chains were typically oriented parallel to the larger thermal expansion coefficient. A birefringence of 0.0046 was attained for the polymer placed on calcite sheet, which is useful for optical retardation devices.

Another way to induce double refraction in PIs is based, as in the case of PVA, on mechanical deformation. In a recent study, two PI structures containing a common cycloaliphatic segment were surface-textured using a complex procedure to verify their applicability as orientation sheets for nematics in liquid crystal display devices [37]. The surface adaptation route involved stretching and/or rubbing. When compared with the pristine PI layers, it was demonstrated that the surface modification produced additional birefringence (examined by refractometry). If only rubbing or stretching was used, the polymer had larger Δ*n* (than the unmodified sample), but did not exceed the values corresponding to the films modified using a two-step technique. The stretching accompanied by pressing led to macromolecular alignment in the bulk of the sample, while rubbing acted only at the superficial layers of the polymer film, thus creating variations in the optical anisotropy. When combining such mechanical deformation stages, it appears that the initial deformation step produced a more significant effect with regard to the generated anisotropy, as quantified by Δ*n*. When the principal step led to larger sample orientation, the next step determined less supplementary orientation. For instance, for PI films that were first rubbed, a higher orientation of chains was attained, which was further accentuated by stretching. For the PIs with more flexible chains, a better response to the imposed deformation was observed (Δ*n* = 0.0022). The optical birefringence was correlated with the morphological anisotropy of the surface features noted during atomic force microscopy scans. A higher azimuthal anchoring energy was achieved, showing an improved interaction between the birefringent PI and the nematic molecules. Such results can be explored in the manufacturing of liquid crystal displays.

An additional route for inducing birefringence is by utilizing photosensitive macromolecular structures and polarized radiations of precise energy. Within this scope, various amounts of an azochromphore were inserted into a PI, and the system was exposed to a UV laser [11]. The presence of the azo-dye determined variations in the aspect of the PI samples, particularly a slight yellowing effect. Moreover, a larger amount of azochromphore was responsible for enhancement of the average refractive index and a considerable increase in the birefringence (measured by means of an Abbe refractometer with polarizing eyepiece). Therefore, for nonirradiated samples, *Δn* varied from 2.2 × 10^−3^ (PI with lowest amount of dye) to 2.8 × 10^−3^ (PI with highest amount of dye). For the UV laser-treated samples, birefringence was considerably enhanced to 0.014 (PI with lowest amount of dye) and 0.0365 (PI with highest amount of dye). This aspect and the created regular surface morphology represent essential factors highlighting these materials as ideal candidates for optical storage devices.

It can be concluded that most PIs, especially those containing aliphatic segments, are not birefringent. Optical anisotropy can be generated in the following cases: (a) preparation of films on substrates having strong anisotropy of the thermal expansion coefficient; (b) mechanical deformation (stretching and/or rubbing); (c) irradiation of PIs doped with photosensitive substances. The molecular ordering is dependent on the PI chain flexibility, as well as on the level at which the deformation force is acting. The order of applied stress seems to have a deep impact on the induced optical anisotropy. Larger birefringence can be accomplished by inserting photo-responsive molecules, followed by exposure to coherent and polarized radiations. The materials must be irradiated at the wavelength at which the embedded substance absorbs in order to induce the desired molecular alignment. Birefringent PI materials are useful for making textured layers for liquid crystal displays, retardation films, and recording media for optical storage devices.

### 4.4. Birefringence of Materials Based on Cellulose Derivatives

Cellulose and its derivatives are very interesting materials that combine biodegradability with excellent film-forming ability, mechanical strength, and optical clarity. This natural polymer has a level of crystallinity, and the orientation of its domains can lead to double refraction [70]. The intrinsic birefringence of cellulose was determined by retardation mapping as 0.09 [53]. Birefringence-derived orientation of the cellulose nanofibers from transparent foils was also emphasized using a less common technique that relied on image investigation of the polarized light micrographs [71]. Thin films were attained using the filtration technique and were wet-stretched; for comparison reasons, randomly oriented samples were attained. Images under polarized light were initially acquired at −45° and +45° with regard to the deformation direction of the solid sample (0°). Upon rotation of the image, acquired at −45° with 90° counterclockwise, two images were obtained, which could be overlapped in a complex picture. Data related to birefringence were computed by examination of the modifications of the intensity of the blue channel in both pictures for all pixels. When the resulting values of birefringence-related parameter were null, this indicated no orientation in the cellulosic sample, whereas, if data were in the intervals of −1 or +1, this indicated an orientation in the orthogonal direction with regard to the deformation direction.

However, cellulose solubility is limited; for this reason, its derivatives have received huge attention. Moreover, when placed in appropriate solvents, certain cellulose ethers and esters gain liquid crystal properties [72,73]. The variation of the birefringence modification with wavelength was studied for some cellulose esters [31]. According to experiments with optical birefringence analyzer, it was reported that cellulose triacetate (CT; with three acetyl groups) presented negative birefringence with ordinary dispersion (i.e., the absolute value of birefringence was lower for higher wavelengths). On the other hand, cellulose acetate propionate (CAP) displayed positive birefringence with extraordinary wavelength dispersion due to the presence of two esters (birefringence increased for larger wavelengths). Thus, at 500 nm, CAP had Δ*n* = 5 × 10^−4^, whereas CT had Δ*n* = −12.4 × 10^−4^. At 750 nm, the birefringence of CAP and CT was 6 × 10^−4^ and −8 × 10^−4^, respectively [74].

Another study dealt with the estimation of Δ*n* of cellulose triacetate using other two techniques [60], namely, multiple-beam Fizeau fringes [75] and the Becke line technique, for estimating the refraction properties in the exterior of fibers parallel and orthogonal to their axis [76]. Both methods led to similar refractive indices, indicating that the analyzed cellulosic sample was isotropic (Δ*n* = 0).

In the case of hydroxypropyl cellulose (HPC) solutions, the channeled spectrum method was applied to evaluate birefringence [77,78]. The data showed that the birefringence magnitude increased with the polymer concentration in solution. At 500 nm, Δ*n* was 0.000102 (30% HPC in water), reaching 0.000104 (45% HPC in water). In addition, at the same concentration, it was found that the type of used solvent influenced the birefringence results. Thus, at 450 nm, the sample in water had Δ*n* = 0.000118, whereas, in methanol, Δ*n* = 0.000122, and, in acetic acid, Δ*n* = 0.000124. Another study [54] described the double refraction near the critical concentration for the anisotropic phase. The data (recorded with an Abbe refractometer) were compared with those attained for other polymers obtained by esterification of HPC with 4-alkoxybenzoic acid having various carbons. For HPC at 46 wt.% in dimethylacetamide, Δ*n* was 0.0061, whereas, for the esterified counterparts at concentrations of 42–44 wt.%, it was demonstrated that, as the length of the alkoxy group increased, the birefringence was reduced from 0.00911 to 0.0022. This information is useful for producing liquid crystal-based devices. The birefringence of HPC was also investigated in a wide concentration range comprising the occurrence of the liquid crystalline phase [79]. For concentrations of 30–35%, Δ*n* exhibited an abrupt increase; since the order parameter did not change much, this was seemingly a biphasic interval. Then, a significant increase in Δ*n* was noted when the liquid crystalline phase appeared. Furthermore, the HPC sample in the cholesteric phase presented a linear increase in Δ*n* with the polymer concentration.

The depicted reports show that the crystalline nature of cellulose is characterized by an intrinsic birefringence, but its low solubility limits its characterization and applicability. Hence, after chemical modification, it has been found that the derivatives of this natural polymer present negative or positive birefringence as a function of the chemical nature of the side-groups. Cellulose ethers such as HPC can form a cholesteric phase in certain solvents, whereby the birefringence varies abruptly until the liquid crystalline phase is formed, which can be expressed as a linear variation. The critical concentration at which the cholesteric phase is reached depends on the type of solvent; hence, the measured birefringence of HPC is also influenced by this factor. Cellulosic materials exhibiting birefringence can be used in optical and photonic devices.

### 4.5. Birefringence of Other Polymer Materials

Birefringence is a phenomenon studied in many other polymer structures. Among engineering plastics, poly(methyl methacrylate) (PMMA) is a synthetic and thermoplastic material of interest in manufacturing optical devices. The relationship between the applied deformation and birefringence of PMMA was reported in [80]. The films were subjected to strain compression, and the optical characterization was performed using a semi-quantitative approach relying on the theory of rubber birefringence. In conditions of reduced strain, the strain-optical coefficient was not affected by temperature in the interval of 20 °C to 80 °C. At temperatures lower than 20 °C, the strain-optical coefficient values became increasingly negative (even when enhancing the frequency). Conversely, the stress-optical coefficient remained unmodified up to 20 °C, before becoming highly negative in the range of 20–80 °C. The change in optical properties under variable heating was ascribed to the rotational isomerism from the ester side-unit. There was a progressive decrease in negative birefringence with temperature change within the −200 °C to 100 °C range. A recent study [81] investigated the possibility to compensate for the birefringence of PMMA (Δ*n* < 0) by combining it with bottlebrush polymers (BPs) of known positive Δ*n*. By blending these polymer structures, it was revealed that the orientation birefringence could reach a null value when the ratio of linear PMMA to PMMA-BP was 73:27 (independent of the strain level). Such control of birefringence by simple mixing is suitable for the production of materials for many optical devices, such as lenses.

Poly(propylene oxide) (PPO) is high-strength thermoplastic material which is advantageous for its good processing. The channeled spectra of this polymer were obtained in benzene solution with a concentration of 30 g/mL [55]. The results evidenced a decrease in Δ*n* with increasing wavelength. At 400 nm, the birefringence was 0.00017, decreasing to 0.00002 at 740 nm. The dispersive parameter of Δ*n* presented a linear increase when the wavelength was enhanced. Its magnitude was around 10^−6^, indicating the good accuracy of birefringence computations.

Polystyrene (PS) is versatile material employed for manufacturing numerous consumer products. A complex analysis of the birefringence phenomenon in PS involved monitoring the Δ*n* variation with stretching ratio, stretching speed, and temperature [56]. As in the case ofPPO, the birefringence decreased when the wavelength ranged from 450 nm to 650 nm. An almost linear dependence was attained between Δ*n* and stretching ratio. The largest birefringence of PS was noted at the biggest stretching level of 8, where Δ*n* = 0.00255. In addition to this, when the stretching speed was modified from 90 to 210 mm/s, the birefringence increased in the interval of 1 × 10^−3^ to 2.3 × 10^−3^. The temperature (52–58 °C) generated a linear decrease in Δ*n* from 2.5 × 10^−3^ to 1.8× 10^−3^. These data are important for making retardation films for implementation in diodes or displays.

On the basis of the above studies, it can be stated that birefringence can be recorded in polymers containing chiral centers in their structure, while those that lack chirality can become birefringent via mechanical deformation. Optical anisotropy of PMMA leads to either positive or negative birefringence in specific temperature conditions, whereas the Δ*n* of PPO or PS remains positive in specific wavelength and temperature intervals. Such transparent and anisotropic materials are of great interest for the development of lenses and retardation films for optical instruments.

## 5. Conclusions and Future Outlook

Optical polymers exhibit certain challenges to designers, mainly regarding a narrow domain of refractive index values, small thermal stability, and reduced optical birefringence. This manuscript described some relevant optical techniques that are useful for the analysis of birefringence in polymer materials, focusing on the working principles and mathematical bases developed to evaluate Δ*n*.

Significant studies on optically birefringent polymers were presented for PVA, PET, PI, cellulose and its derivatives, PMMA, PPO, and PS. The birefringence was intrinsic in a few cases, but most materials required the presence of an external force (tensile, shear, compression, bending, or twisting stress) to acquire double refraction characteristics. It was found that the nonbirefringent character of PVA changes after mechanical stressing, freezing/thawing treatment, exposure to UV or gamma radiation, action of acoustic waves, and dissolution in a solvent of distinct thermodynamic quality. As a function of the incoming radiation energy, Δ*n* of the exposed films might be higher or lower in comparison to the unexposed samples. The composition of solvent mixture seems to have a larger impact on Δ*n* than the crystallinity level, degree of polymerization, and concentration. When mixed with drugs, dyes, or other polymers, PVA displays significant modification of birefringence. Insertion of iodine, for instance, is advantageous for facilitating chain slippage during mechanical stress; hence, the registered Δ*n* is larger for PVA/iodine films. Investigations on PET showed that this material has a dispersive birefringence; moreover, under biaxial stretching, the variation of Δ*n* with imposed stress has zones characterized by distinct slopes. Within a certain experimental temperature range, PET displays different orientation of the amorphous or crystalline zones, which was reflected by an increase in Δ*n*. Studies on PIs revealed that birefringence is not observed when charge transfer complex interactions are lowered by the presence of aliphatic segments in the main chain. Birefringent PI materials are attained when the films are casted on substrates with anisotropy of the thermal expansion coefficient, mechanical deformation, and exposure to laser upon the addition of photosensitive molecules in PI. The order of applied stress seems to have a deep impact on the induced optical anisotropy, whereas higher Δ*n* is noted in PI-based materials in the case of photogenerated birefringence in comparison to the other two situations (substrate anisotropy and stress application). The crystalline nature of cellulose is characterized by an intrinsic birefringence. Its derivatives have negative or positive birefringence as a function of the chemical procedure used to compensate for cellulose’s lack of solubility. For example, CT displays negative Δ*n*, whereas CAP and HPC present positive values for this parameter. Moreover, HPC is able to render a cholesteric phase in certain solvents, and the level of molecular ordering is affected by the type of dissolution medium. Thus, the critical concentration of the cholesteric phase differs according to the solvent features, and the level of acquired birefringence is distinct. On the other hand, Δ*n* can be found in polymers containing chiral centers in their structure, while those that lack chirality can become birefringent via mechanical deformation. Optical anisotropy of PMMA can yield positive or negative Δ*n* within certain temperature intervals. Birefringence of PPO or PS is positive regardless of the light wavelength and temperature. Mechanical deformation leads to orientational birefringence for all polymer systems. The described materials have particular applicability in various domains, including liquid crystal displays, diodes, airspace components, and optical filters.

The future of this scientific domain resides in discovering original approaches to control birefringence in polymers and adapt their optical performance to the application needs. Some research paths need further exploration, such as the examination of birefringence variation in blends of isotropic/anisotropic polymers or in textured polymer films using complex approaches. Such new directions are expected to contribute to certain considerable advancements in establishing methods to tune both the morphology and the optical performance of materials, as well as expand their functionality when implemented in modern optical technologies.

## Figures and Tables

**Table 1:** The values of birefringence induced by various methods for some polymer materials.

Polymer Material	Δ*n*	Characterization Method	Orientation Technique/Conditions	Reference
Polyvinyl alcohol (PVA)	0.0311	Babinet compensator	Stretching	[39]
	0.0262	Polarizing ellipse method	Stretching degree of 2.6	[36]
	0.0047	Babinet compensator	Gamma exposure, Stretching ratio of 1.6	[40]
	0.0183	Babinet compensator	Microwave irradiation, stretching ratio of 3	[41]
PVA/pyridinium ylid	0.0320	Polarizing microscope	Uniaxial stretching, stretching degree of 0.35	[42]
PVA/phthalazinium ylid	0.0254	Babinet compensator	Stretching degree of 5.5, gentile heating	[43]
PVA/cycloimmonium ylids	0.0275	Babinet compensator	Stretching ratio of 5	[44]
PVA/donepezil	0.0250	Babinet compensator	Stretching degree of 1.4	[45]
PVA/Congo red dye	0.0800	Babinet compensator	Stretching degree of 3	[46]
Polyethylene terephthalate	0.0550	Channeled spectra	589 nm	[47]
	0.2069	Ziess polarizing microscope	Hot multistage drawing, 130 °C, drawing ratio 6.24	[48]
	0.0200	Lab-made device	Biaxial stretching, 95 °C	[49]
	0.0450	Mueller matrix ellipsometry	Biaxial stretching, 23 °C	[50]
Polyimides (PIs)	0.0400	Polarizing ellipse	Conditions of synthesis, aromatic structure	[51]
	0.0170	Polarizing ellipse	Semi-aliphatic structure	[52]
	0.0040	Polarizing ellipse	Fully aliphatic structure	[52]
	0.0022	Refractometry method	Rubbed and stretched	[37]
	0.0365	Refractometry method	Azo-dye, UV laser	[11]
Cellulose	0.0900	Retardation mapping	Ambiental	[53]
Cellulose triacetate	0.0005	Optical birefringence analyzer	Stretching, 500 nm	[31]
Cellulose acetate propionate	−0.0012	Optical birefringence analyzer	Stretching, 500 nm	[31]
Hydroxypropyl cellulose	0.0061	Refractometry method	Liquid crystal	[54]
Poly (propylene oxide)	0.00017	Channeled spectra	Solution in benzene, 400 nm	[55]
Polystyrene	0.00255	Optical interference method	Stretching ratio of 8	[56]

## Data Availability

Not applicable.
